# No one size fits all—the development of a theory-driven intervention to increase in-hospital mobility: the “WALK-FOR” study

**DOI:** 10.1186/s12877-018-0778-3

**Published:** 2018-04-13

**Authors:** Anna Zisberg, Maayan Agmon, Nurit Gur-Yaish, Debbie Rand, Yehudit Hayat, Efrat Gil, the WALK-FOR team

**Affiliations:** 1 The Cheryl Spencer Department of Nursing, Faculty of Social Welfare and Health Studies, University of Haifa, Haifa, Israel; 2 The Center for Research and Study of Aging, Faculty of Social Welfare and Health Studies, University of Haifa, Haifa, Israel; 3 Department of Occupational Therapy, School of Health Professions, Sackler School of Medicine, Tel Aviv University, Tel Aviv, Israel; 4 Department of Nursing, HaEmek Medical Center, Afula, Israel; 5 Clalit Health Services, Haifa, Israel

**Keywords:** Older adults, Hospitalization, Mobility, Theory-driven intervention, Functional decline, Step count

## Abstract

**Background:**

There is growing evidence that mobility interventions can increase in-hospital mobility and prevent hospitalization-associated functional decline among older adults. However, implementing such interventions is challenging, mainly due to site-specific constraints and limited resources. The Systems Engineering Initiative for Patient Safety (SEIPS 2.0) model has the potential to guide a sustainable, site-tailored mobility intervention. Thus, the aim of the current study is to demonstrate an adaptation process guided by the SEIPS 2.0 model to articulate site-specific, culturally based interventions to improve in-hospital mobility among older adults.

**Methods:**

Six consecutive phases addressed each of the model’s elements in the research setting. Phase-1 aimed to determine a measurable outcome: steps/d, measured with accelerometers, associated with functional decline. Phase-2 included interviews with key persons in leadership positions in the hospital to explore organizational factors affecting in-hospital mobility. Phases-3 and 4 aimed to identify attitudes, knowledge, barriers, and current behaviors of medical staff (*n* = 116) and patients (*n* = 203) related to patient mobility. Phase-5 included four focus-groups with unit staff aimed at developing an action plan while adapting existing intervention strategies to site needs. Phase-6 relied on a steering committee that developed intervention-adaptation and implementation plans.

**Results:**

Nine hundred steps/d was defined as the intervention outcome. 40% of patients walked fewer than 900 steps/d regardless of capability. Assessing or promoting mobility did not exist as a separate task and thus was routinely overlooked. Several barriers to patients’ mobility were identified, specifically limited knowledge of practical aspects of mobility. Consequently, staff adopted practical steps to address them. Nurses were designated to assess mobility, and nursing assistants to support mobility. Mobility was defined as a quality indicator to be documented in electronic medical records and closely supervised by hospital and unit management. Preliminary analyses of the “Walk FOR” protocol demonstrates its’ ability to reduce barriers, to re-shape staff attitudes and knowledge, and to increase in-hospital mobility of older adults.

**Conclusions:**

The SEIPS-2.0 model can serve as a useful guide for implementing a site-tailored comprehensive mobility intervention. This process, which relies on local resources, may promise sustainable practice change that may support early effective rehabilitation and recovery.

**Electronic supplementary material:**

The online version of this article (10.1186/s12877-018-0778-3) contains supplementary material, which is available to authorized users.

## Background

In-hospital mobility is described as one of the leading modifiable factors which may prevent in-hospital functional decline among older adults with acute illness [1, 2]. During hospitalization, mobility is often limited or reduced to minimal ambulation, such as bed-to-chair transfers [3–5]. Patients have been reported to spend 57 to 83% of their hospital stay lying in bed [5]. For hospitalized older adults, each day of immobility is associated with approximately 1.5 to 3% reduction in muscle mass and a 5% loss of muscle strength [6, 7], which results in longer hospital stay [8], functional decline [5, 7, 9], and limited social participation even 2 years after discharge [10].

Several interventions were developed aiming to improve in-hospital mobility and prevent functional decline. For example, the “Move to Improve” intervention demonstrated increased levels of mobility in intensive care units (ICUs) [11], and MOVIN, a nurse-driven intervention, demonstrated its efficacy in increasing mobility and mobility documentation in a pilot study [12]. A randomized clinical trial by Brown and colleagues showed that improved mobility during hospitalization can reduce functional decline in 1 month post discharge [13]. Despite these positive findings, the generalizability of these interventions to different sites or other cultures with different healthcare systems is yet to be determined. To bridge the gap between the potential contribution of these interventions and site-specific requirements and considerations, a well-grounded yet flexible model is required. The aim of the current paper is to demonstrate the process of adapting a human factors framework, the Systems Engineering Initiative for Patient Safety (SEIPS 2.0) [14], as a guided model to articulate a site-specific, culturally based intervention to improve in-hospital mobility in older adults.

### SEIPS 2.0 model description

SEIPS 2.0 is based on Donabedian’s [15] structure-process-outcome model, which postulates that the human factor is critical to understanding every health interaction and that its role should be explored and considered when designing and analyzing health interventions. The model comprises three consecutive arms: the work system, which produces work processes, which in turn lead to outcomes. The work-system arm comprises six interacting components: persons, tasks, tools and technologies, organization, internal environment, and external environment. The persons, who serve as active agents, are in the center of the work-system arm and constantly interact with the other five components. Moreover, the model incorporates three major principles: configuration, engagement, and adaptation. Configuration is the interrelationship between persons within a specific network given its hierarchical and interactive nature within each model domain and between the domains. Engagement expresses the idea that all persons are involved separately and collectively within a specific health-related activity. Adaptation conveys the dynamic nature of the health system and emphasizes the potential effect of each factor on the whole system when a new practice is introduced [14]. Thus the model emphasizes the importance of mapping site-specific strengths and limitations prior to introducing and implementing existing interventions or designing new ones.

### Defining the SEIPS 2.0 concepts in the context of in-hospital mobility

The SEIPS 2.0 model postulates that well-defined outcomes are crucial to the implementation of any intervention. In the case of in-hospital mobility, well-defined standards of care did not exist before we began this process [13]; thus, it was the preliminary goal of our project. The next step in adapting the SEIPS 2.0 model to our project was to operationalize each of its concepts in the context of the desired outcome: recommended level of mobility (steps/d). According to the SEIPS 2.0 model, mobility, like all hospital outcomes, is a complex concept influenced by specific work systems and processes. The process of in-hospital mobility is central to understanding professional standards and norms related to mobility while mapping each collaborator’s (i.e., patient’s, staff member’s) role and contribution. It includes understanding how, by whom, and under what conditions mobility is initiated or suppressed, documented, communicated, and reported. Relying on the model, the process of in-hospital mobility depends on the work system comprising six interacting components: the person is the central concept, encompassing patients, their families, and the medical team. To this end, their preferences, goals, needs, knowledge, and attitudes should be explored. Tasks are defined by the subjective difficulties, complexity, ambiguity, and sequences applied by “persons” with respect to in-hospital mobility, and objective measurable characteristics, that is, distance or step count. SEIPS 2.0 led us to account for the availability of tools, technology, and a physical environment to support the mobility task. Finally, organizational factors (internal: unit level; external: hospital level) were categorized as declared goals, policies, documentation, written and spoken rules, and procedures related to patients’ in-hospital mobility.

## Methods

### Phase 1: Defining the outcome

In a prospective cohort study, we recruited 203 older adults hospitalized in two internal medicine units at an academic medical center in northern Israel between October 1, 2015, and December 31, 2015. Patients with substantial cognitive impairment, unable to ambulate with or without an assistive device 2 weeks before hospitalization, hospitalized due to disabling diagnosis (severe CVA, need for ICU care), or admitted for end-of-life care were not included. Within 24 h, participants’ functional, cognitive, and emotional status was assessed, as well as their attitude toward in-hospital mobility and their intention to be mobile while hospitalized. Actual mobility was assessed by daily step count using accelerometers (Actical) [16] worn from 24 to 72 h based on patient’s length of stay. Findings from this phase were used to define mobility recommendations and thus served as the intervention’s desired outcome.

### Phase 2: Understanding in-hospital mobility through the lens of the SEIPS 2.0 model

The observation and interview guideline comprises questions from each of the model’s domains as well as the three principles of configuration, engagement, and adaptation. Example questions are: “How would you define in-hospital mobility?” (identification of the task), “In your unit who are the persons responsible for patients’ mobility?” (identification of key persons), “At the hospital level, what is the policy towards mobility?” (identification of the internal environment), “What are the barriers to patients’ mobility?” (identification of tools, internal environment), “Who can assist with patients’ mobility?” (identification of key persons), “If patients need assistance with mobility, do you have enough aids for this purpose?” (tools), and “Do you document mobility, and if so, how?” (identification of internal environment). Full interview guide is available in the supplementary material (see: Additional file 1). The observations focused on the physical hospital environment, reviewing written protocols, patients’ records, and available equipment. Eleven interviews with key persons in leadership positions—head nurse of the hospital, deputy nurse, two unit head nurses and their deputies, four physicians (department heads and their deputies), head of physical therapy in the hospital and study units—were conducted to explore organizational factors affecting in-hospital mobility. Themes from the qualitative exploration were extracted and organized according to the model components.

### Phase 3: Identifying medical staff attitudes, knowledge, barriers, and current behaviors related to patients’ mobility

All staff working in two internal medical units (*N* = 116)—nurses, nurse’s aides (NAs), physical therapists (PTs), and medical doctors (MDs)—were asked to complete a modified version of the Barriers to Early Mobility of Hospitalized General Medicine Patients questionnaire [3]. The instrument consists of items assessing knowledge, attitude, and barriers perceived by the team. In the current version, 23 items in 3 subscales of the measure had an acceptable reliability (see Table 1). The lower the score, the more perceived barriers across all dimensions. In addition, we asked participants their opinion regarding which sectors are responsible for promoting in-hospital mobility. Staff participants also reported demographic and occupational characteristics. The self-report survey was conducted anonymously.

**Table 1:** Descriptive statistics (mean ± sd) and comparisons of healthcare staff’s knowledge, attitudes, and behaviors with respect to patient mobility in the study hospital units

	RN	NA	MD	PT	Cronbach’s ɑ	*F*
Knowledge (1–4)	2.45 ± .36	2.67 ± .56	2.54 ± .58	3.57 ± .35	0.76	30.8**
Attitudes (1–4)	2.53 ± .48	2.68 ± .51	2.61 ± .45	3.00 ± .45	0.60	4.44**
Behaviors (1–4)	2.23 ± .37	2.18 ± .47	2.64 ± .49	2.65 ± .39	0.73	6.33**
Total score (1–4)	2.44 ± .25	2.46 ± .34	2.61 ± .32	2.99 ± .25	0.77	18.22**

Abbreviations*: RN:* registered nurse; *NA:* nurse’s aide, *MD:* medical doctor; *PT* physical therapist

### Phase 4: Identifying patient’s baseline attitude, knowledge, and barriers regarding in-hospital mobility as well as actual in-hospital mobility

Using the same sample described in phase 1, we assessed participants’ attitudes toward in-hospital mobility using a dedicated 6-item scale with established reliability and validity [17].

### Phase 5: Developing an action plan

To achieve this goal, we led four focus groups with units’ staff aiming to adapt existing interventions’ strategies to site needs. The focus groups were conducted to define strategies to improve mobility and to articulate policy to support mobility practice. Each focus group began by introducing findings from staff and patient surveys (phases 3 and 4) following results for actual patient mobility and its association with patients’ functional outcomes [18]. Staff members were asked to comment on the findings and to offer practical strategies for improving mobility. To facilitate the process, we introduced material from other successful interventions [12, 19]. Staff members were asked to evaluate the potential adaptability of each potential idea based on their site-specific culture and resources.

### Phase 6: Intervention adaptation and implementation

Based on phases 2 through 4, where barriers to in-hospital mobility were identified, and phase 5, where potential directions for intervention where weighted by multidisciplinary teams, a steering committee designed the ‘road map,’ which includes three elements: (a) actions to be taken and modes of implementation, (b) responsibilities and designated persons to promote various intervention elements, and (c) time frame and sequence of implementing the intervention in the unit.

## Results

### Phase 1: Defining the outcome

This phase involved measuring actual levels of in-hospital mobility and articulating recommendations for desired mobility levels by linking daily step counts to functional outcomes. Despite the large variation in steps/d (from 0 to 8111), 900 steps and above was identified as a mobility level which prevented a clinically meaningful functional decline (5 points on the Modified Barthel ADL Index) in the vast majority of the study participants [18].

### Phase 2: Findings from qualitative interviews and observations

**Fig. 1:**
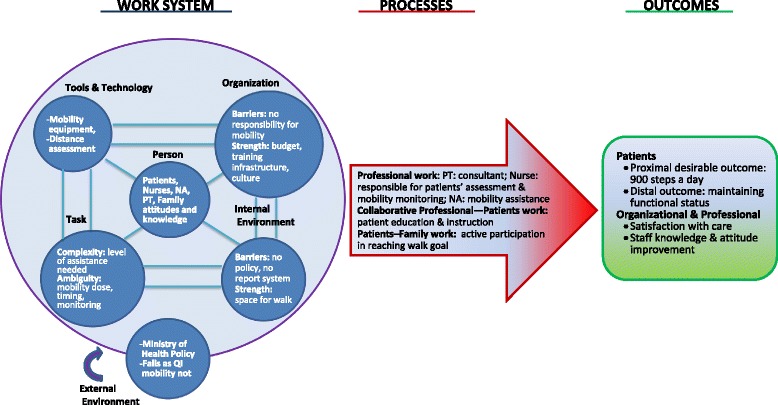
SEIPS 2.0 adaptation for in-hospital mobility intervention. According to the SEIPS 2.0 model, mobility, like all hospital *outcomes*, influenced by specific *work systems* and *processes*. The process of in-hospital mobility conducted by “Professional work”, “Collaborative Professional” and “Patients–Family work” includes understanding how, by whom, and under what conditions mobility is initiated or suppressed, documented, communicated, and reported. The *process* of in-hospital mobility depends on the *Work system* comprising six interacting components: the *person*, encompassing patients, their families, and the medical team comprising of their preferences, goals, needs, knowledge, and attitudes; the *task* comprising difficulties, complexity, ambiguity, and sequences as well as measurable characteristics (distance, step count); *tools & technology*– mobility equipment, distance assessment; *organizational factors* – including barriers & strength; *environment* (internal: unit level; external: hospital level) – goals, policies, documentation, written and spoken rules, and procedures related to patients’ in-hospital mobility.

Based on these interviews, themes were extracted according to the model aspects and divided into the six working-system domains (see Fig. 1). *Persons* were divided into an active agent, who “performs some or all health-related work activity,” and a co-agent, defined as an “indirect or passive contributor” [14]. Active agents included patients treated in the hospital, their relatives, and the healthcare team comprising nurses, NAs, and PTs; MDs were defined as co-agents. In different settings, occupational therapists (OTs) can be involved as well; however, in our setting they were not identified as relevant to patients’ mobility. Defining the task of mobility was challenging, since different sectors defined mobility differently. For example, nurses defined mobility as transfers from bed to chair, some PTs considered stepping in one spot to be a sufficient level of mobility, and MDs saw the definition as irrelevant since they considered most of the patients to be incapable of walking safely. Moreover, there were differences in mobility policies between the units: in one unit, most patients were defined as “need[ing] to stay in bed” for the first 24 h of hospitalization, while no clear policy was identified in the other. In accordance, the interviews revealed that mobility was perceived as a complex task requiring different resources and regimens according to the patient’s status. When asked about *tools* for mobility support, the team mentioned a lack of resources of mobility aids, especially the limited walkers available to use. Moreover, the translation of mobility level into measurable distance was not clear. Analysis of *organizational* factors demonstrated several strengths that may support practice and policy changes. For example, a tradition of strong teamwork and willingness to adopt new practices were described by the teams. In addition, a well-structured training system for different purposes exists in the hospital, as well as management willingness to invest money to support the proposed changes in practice. Along with these strengths, several limitations arose. It was not clear who was responsible for “prescribing” mobility and who was responsible for directly assisting patients. Exploring the Ministry of Health regulations and policies, that is, the *external environment,* revealed a formal document that defines the nurse as the main person responsible for patients’ mobility while emphasizing the importance of patients’ mobility. Nonetheless, this regulation was not assimilated into daily routines, and staff members were not familiar with it. At the same time, we found fall prevention to be an important quality indicator (QI) that is widely implemented and closely monitored. Consequently, mobility was compromised for the sake of fall prevention. Observations of the *internal environment* showed enough open space within the department’s halls to enable free mobility. The internal medical records did not support mobility documentation or other mobility protocols. Mapping the six work-system interacting components enabled us to distinguish between these aspects and thus articulate our next steps.

### Phase 3: Identifying medical staff attitudes, knowledge, barriers, and current behaviors related to patients’ mobility

The staff-member response rate was relatively high (78.3%), with the highest representation of PTs (95%) and nurses (82.7%) (response rates for MDs and NAs were 76.9% and 50%, respectively). Comparisons of knowledge, attitudes, and behaviors regarding in-hospital mobility across professions showed that PTs had the highest knowledge and the most positive attitudes toward mobility. The rest of the staff showed relatively poor knowledge and negative attitudes, without significant differences between sectors (see Table 1).

The behavioral aspects, focusing mainly on the identification of barriers to mobility, showed a slightly different pattern: MDs and PTs showed lower barriers to mobility compared to nurses and NAs.

The items (mean ± SD scores out of a maximum 4 points) reflecting the highest perceived barriers for all healthcare providers were as follows: “The staff is not adequate to mobilize inpatients on my unit” (2.1 ± .8), “We don’t have the proper equipment to mobilize my inpatients” (1.6 ± .9), and “The mobility level of my inpatients is regularly discussed between the patient’s healthcare providers” (1.8 ± .8). In addition, beside PTs, all other healthcare providers rated the item reflecting their knowledge of safe mobilization relatively low (“I have received training on how to safely mobilize my inpatients” [2.2 ± 1.0]). Finally, the item with the largest differences in mean scores between nurses and NAs was related to NAs disagreeing with not having time to mobilize their inpatients during their shift/workday (1.6 for RNs vs. 2.9 for NAs). Consensus emerged among healthcare providers that inpatient mobility efforts should be a multidisciplinary endeavor including all health-provider sectors as well as family and patients. However, while PTs were rated as 100% responsible for in-hospital patients’ mobility, patients themselves and family members were rated around 80%, nurses and NAs around 70%, and MDs less than 50%.

### Phase 4: Identifying patient’s baseline attitude, knowledge, and barriers regarding in-hospital mobility as well as actual in-hospital mobility

Of the 203 enrolled participants, 93% (189) completed the data-collection procedure including in-hospital mobility measurement. Most participants had positive attitudes (3.36 ± .97, on 1–5 scale), with 65% (131) expressing above-neutral values. However, 46.5% agreed to some extent with the statement “I need to stay in the bed when I’m sick,” and 39.6% disagreed with the statement that “walking in the hospital will help me maintain my pre-hospital function.” Of the participants, 37.6% (71) walked fewer than 900 steps (identified as a minimum desirable threshold [18]); low walkers walked on average 374 steps (±252). Negative-to-neutral attitudes toward mobility at time of hospital admission were significantly related to walking fewer than 900 steps during hospitalization, and the odds of low mobility were 3.22 (95% CI: 1.24–8.44) times higher for patients holding negative-to-neutral attitudes than for those with positive attitudes, even when controlling for objective mobility ability on admission, severity of illness, length of stay, cognitive function, age, and premorbid mobility level.

### Phase 5: Developing an action plan

Four focus groups were conducted to address barriers to mobility and to articulate the intervention protocol. The first group included all staff members from the units and from the hospital headquarters. In this group, findings from the previous phases were presented, including qualitative themes, knowledge of and attitudes toward mobility, actual level of mobility, and its association with functional outcomes. Moreover, interventions implemented in different settings to improve mobility were presented. The participants were asked to comment on these findings and to suggest ways to improve mobility levels. The predominant themes that arose were lack of knowledge regarding walking safety assessment, disagreement regarding who is responsible for patients’ mobility, and lack of resources.

A second focus group was then conducted by the study’s researchers and included local leaders: two unit head nurses, the head of PT, and four nurse supervisors. The aims of this group were to articulate the policy toward mobility, to share responsibilities between sectors, and to address the best way to increase knowledge on mobility. This group led to defining the nurse as the main person responsible for evaluating mobility at admission. If needed, she can consult with a PT. It was agreed that 900 steps/d is the desired outcome and that each nurse should follow all her patients with this goal in mind. In addition, based on the NAs job description and availability, they were identified as a sector which can actually support mobility. Therefore, the responsibility for walking specific patients within specific time slots, was given to one of the personal working during each shift. Time slots between 11 am to 12 pm and 5 pm to 6 pm were defined by the staff as the preferable times for assisting mobility. No additional workforce was needed. Interestingly, preliminary analysis revealed that only 20% of patients (2–4 patients per day) required walking assistance from the NA (the remaining patients could walk independently or with the help of their family). To closely follow up on the mobility, recording mobility should be documented by hand and electronically in the electronic medical records (EMRs). To improve staff members’ knowledge, two strategies were offered: (1) an online tutorial, and (2) face-to-face training by PTs.

Two additional focus groups were led by the head nurse and included nurses and NAs. In these meetings the head nurse presented the protocols for mobility and defined mobility as a local QI that will be daily monitored by her team. They discussed ways to increase patients’ and families’ awareness of mobility since family members were identified by the team as potential resources to assist patients and support mobility. Ideas based on the “Move to Improve” intervention were discussed, and the following ways to increase awareness to mobility were decided on. The required distance of 900 steps (i.e., 500 m) will be marked on the department walls, a brochure regarding the importance of hospital mobility will be provided to families on admission, and a short video-clip regarding the importance of mobility will be shown on the TV screens within the unit. Nurses asked to purchase more walkers and offered ways to guard them against theft.

Following these four consecutive group meetings, a 1-month time frame was proposed for introducing all elements of the intervention.

### Phase 6: Intervention adaptation and implementation

The intervention stages were developed simultaneously by dedicated team members assigned to each task in phase 5. One group, comprising a researcher, a PT, and nurses, designed educational materials: an online educational module for on-the-job theoretical training (partially adapted from the MOVE ON project [19]), followed by bedside training by PTs. Another group included nurses with expertise in informatics and medical records. This group designed electronic reports of mobility assessments, after receiving institutional approval. After the module was pilot-tested by the team for feasibility and clarity, it was implemented as mandatory to report mobility within the study units. An additional team worked with the hospital engineers and purchasing department to design walking trails, mark them appropriately, and purchase extra walkers for each study unit. Finally, a bilingual team (Hebrew-Russian; Hebrew-Arabic) of RNs and PTs designed brochures and posters for patients and families that included recommendations and helpful tips for maintaining mobility (partially adapted from MOVE ON) [19]. All materials were carefully adapted for the unique cultural background of patients in Israel and piloted for clarity with medical teams as well as patients in each language. The head nurse in each unit conducted team meetings to introduce all designed intervention materials, discussed how each would be adopted and incorporated into the daily workflow, and scheduled training of staff members. In addition, the teams on each unit finalized the workflow related to assessing patients’ mobility, evaluating mobility levels, and reporting this data in EMRs. Finally, a structured supervision process was articulated and included daily reviews of EMRs and weekly discussions of the mobility protocol implementation.

### Preliminary results

A. *Staff members’ outcomes.* Three months after implementing the “Walk FOR” protocol, 83 staff members were given the modified version of the Barriers to Early Mobility of Hospitalized General Medicine Patients questionnaire. Fifty-seven 69% of them participated in phase 3 of the study and filled in this questionnaire the second time. The main findings indicate that the “Walk FOR” protocol improved knowledge (F = 8.36_(56,1),_ *p* = 0.005), behavior (F = 72.16_(56,1)_, *p* < 0.001), and attitudes (F = 4.68_(56,1)_, *p* = 0.035) of the hospital staff, toward in-patients’ mobility. The most significant findings were the improvement of knowledge and behavior among nursing aides and nurses.
B. *Patients’ outcomes.* We evaluated the impact of “Walk FOR” by comparing data from three sources before versus after protocol implementation:

1. Staff encouragement: Only 32 (17%) patients versus 157 (84%) reported to receive walking encouragement (*p* < 0.001, *χ*^*2*^ = 121.0).
2. Patients’ attitudes: 92 (48%) patients versus 107 (57%) patients responded positively to the phrase “I believe that increasing in-hospital mobility will improve my recovery” (*p* < 0.001, *χ*^*2*^ = 32.8).
3. Level of patients’ mobility: Patients walked double the number of steps per day after protocol implementation (1243 steps versus 2356 steps, *p* < 0.001): and the number of patients who walked more than 900 steps per day after the protocol implementation was 1.4 times higher than before (87% vs. 61%, respectively, *p* < 0.001).

## Discussion

The current study demonstrates a process for developing an intervention aimed at improving in-hospital mobility while relying on a human factor system model which defines the main concept (i.e., mobility) as a dynamic one shaped by socio-technical and human healthcare factors. Findings from our study support in-hospital mobility as a dynamic, locally embedded concept. Although the existing literature provides a variety of strategies and tools to improve inpatients’ mobility [3, 12, 13, 19–22], based on the SEIPS 2.0 model’s etiology, an existing intervention could not be easily incorporated since this process required a site-specific analysis and configuration.

The first step in designing the intervention was to define its outcome: desired level of mobility. In our sample, as shown earlier, there was disagreement regarding the definition of inpatient mobility, and different sectors had their own measurable outcomes such as time spent in a sitting position, standing in place, or walking to the toilet. Providing an evidence-based operational definition (900 steps/d) helped the staff reach agreement on an interdisciplinary measurable goal for the desired practice that will be implemented during the intervention.

The second step, according to the SEIPS 2.0 model, was to map the work system (organization, internal/external environment, tools, tasks, and persons) to reveal site-specific barriers and strengths affecting mobility practices and their interrelationships. The main *organizational* barriers were identified. Patients’ lack of awareness of the importance of mobility during hospitalization was the main barrier which was found to be highly associated with the actual low level of mobility in our sample; this may stem from local practice and policy. An in-depth exploration revealed that the practice of fall prevention, which includes communicating with patients about the risks and dangers of falls and extra precautions while walking, with no emphasis on the importance of mobility, was one of the main factors contributing to patients’ attitudes. Along the same lines, reviewing Ministry of Health regulations, which constitute the *external environment,* demonstrated that fall risk assessment and fall prevention are important QIs whereas level of mobility is not. These findings are supported by those of Hoyer and colleagues [3], who demonstrated that incorporating mobility levels as a QI may increase mobility levels from 43% to 70% on the Johns Hopkins mobility scale. These insights shaped our intervention by emphasizing the need to incorporate mobility as a QI that will guide practices toward mobility.

Recognizing all *persons* engaged in the mobility process was an additional aspect of our inquiry. The mapping process was based on interviews and observations. This triangulated process enabled us to reveal the full picture. In our setting, different people are engaged in and as such may promote or hinder in-hospital mobility. For example, during daytime 90% of the patients were accompanied by family members, similar to other studies from Israel [23]. Therefore, family members were identified by the staff as potential facilitators of mobility. However, due to staff members’ lack of knowledge, families received no guidance on the importance of mobility or on how to promote mobility; consequently, patients stayed in bed. To provide such guidance, various educational activities were developed and offered, such as instructional videos displayed on televisions in the units and instructional brochures. The NAs were another sector identified as potential support for mobility. Following this finding, NAs were actively invited to design the intervention, and since the staff survey revealed that the NAs’ knowledge of patient mobility is very limited, clear instructions for facilitating mobility and adding this task to their daily schedule were incorporated into the intervention plan. Interestingly, neither family members nor NAs were identified in other mobility-promotion interventions as potential resources to support mobility. In other interventions they mostly relied on either external resources [13] or local resources such as nurses [12, 20] and volunteers [21]. Needless to say, NAs and family members are highly valuable support for patient’s mobility in healthcare settings where the nurse-to-patient ratio is low, as in Israel [24].

*Task* is another important aspect of the work system. To address current practice regarding the task, we sought to understand how and under which conditions mobility is performed. Preliminary observations of unit routines indicated that patients’ practice of walking did not take place, other than sporadic walks to the toilet; however, the in-depth interviews with key personnel revealed a more complex picture. For example, we found that different policies exist in two units in the same hospital: the need for a doctor’s prescription for mobility in one unit and the absence of this protocol in another. The difficulty of identifying which staff member is responsible for mobility and for promoting patient mobility supports previous studies from around the world [25]. Mobility, formerly a part of nursing responsibilities, is almost excluded from the nursing task list in recent decades [26, 27]. In addition, in-depth interviews and observations revealed that the mobility task does not appear in any sector task list. Even more striking was the fact that the EMR does not include the option to document mobility.

Relying on multiple-source analyses based on the work systems and the process toward mobility revealed that before the “Work FOR” protocol there was no clear procedure for evaluating, reporting, supporting, and promoting patients’ walking. Even for patients who could walk independently, mobility was deemphasized. This was due to the system’s bureaucracy, lack of protocols, and lack of a designated sector responsible for promoting mobility. To address this gap, we structured a clear process, guided by written protocols, to facilitate patients’ mobility. This process was articulated by the local teams while considering their workload, work routines, and responsibilities. Staff team expressed their need for support and supervision from management and a constant need for process evaluation and feedback. Indeed, the evidence supports the strength of designing an intervention in a bottom-up process while collaborating with team members [28]. Note that a constant feedback-loop mechanism was created through the process and that NAs have expressed excitement about their involvement in the future intervention.

This study is the first to develop a model-based, site-tailored intervention for promoting in-hospital mobility; however, this process had several limitations. According to the model, all persons related to the task should be included in the analysis phase as a source of information and, consequently, involved in the intervention. In the current study, information about patients’ attitudes toward mobility was collected directly; however, information about families’ attitudes was collected indirectly, via the patients. Since families are a potential resource to support mobility, future development should focus more on the families’ point of view and consider them at an earlier stage. The intervention was designed for a relatively homogenous population of hospitalized older adults who are able to walk and are cognitively intact and therefore might not generalize to lower functioning patients. Our preliminary findings demonstrate that “Walk FOR” protocol is feasible to use in hospital internal units and its implementation can improve the knowledge, attitude and behavior of staff, which further results in significantly improved patient outcomes.

## Conclusion

The SEIPS 2.0 model offers a comprehensive and flexible framework for developing a site-specific intervention to promote mobility. The model guides an in-depth exploration considering all persons and processes within a specific network while relying on local resources. Adopting this model may help create a sustainable intervention to significantly change clinical practice that promotes mobility and decreases negative hospital-associated patient outcomes.

## Additional file

Additional file 1: Interview guide for qualitative interviews in WALK-FOR study. (DOCX 15 kb)

## Abbreviations

ADL: Activities of daily living
EMR: Electronic medical record
ICU: Intensive care unit
MD: Medical doctor
NA: Nurse’s aide
OT: Occupational therapist
PT: Physical therapist
QI: Quality indicator
RN: Registered nurse
