# Supplementary material for: No one size fits all—the development of a theory-driven intervention to increase in-hospital mobility: the “WALK-FOR” study
Source: BMC Geriatr. 2018 Apr 13;18:91. doi: 10.1186/s12877-018-0778-3 (PMC5899407; doi:10.1186/s12877-018-0778-3)
Supplement: Supplementary file 1 — Interview guide for qualitative interviews in WALK-FOR study. (DOCX 15 kb) [file 12877_2018_778_MOESM1_ESM.docx]

Additional file 1: Interview guide for qualitative interviews in WALK-FOR study:

- What is your professional background?
- What is your current position in the hospital?
- How long have you held this position?
- Please describe your responsibilities in the hospital and in the department/unit:
- Please describe the functional level of the patients in your department/unit:
  - Can you provide a rough estimation of how many of them were independent in ADL before hospitalization?
  - How many can ambulate with or without assistance during their hospital stay?
- Do you have any official policy regarding patients’ mobility in your ward? If so, what is the content of this policy?
- Do you evaluate patients’ mobility on a regular basis? How?
- How do you define patients’ mobility within the hospital? (What will be considered as a minimum requirement for you to define a patient as mobile?)
- What are your hospital/unit mobility goals?
- What are the barriers to patients’ mobility?
- In cases that patients need assistance with mobility, who can help? And do you have enough aids for this purpose?
- Is there any hospital level policy/ recommendations regarding levels of patients’ mobility?
- Do you discuss patients’ mobility during your team meetings?
- Who is in charge of patients’ mobility in your hospital/unit?
- Do you have any personal responsibilities regarding patients’ mobility?
- Do you think that there is more to do about patients’ in hospital mobility? If so, what? Who should promote that?

*Thank you for your participation in this project*
